# Supplementary material for: WordCommentsAnalyzer: A windows software tool for qualitative research
Source: F1000Res. 2018 Sep 4;7:536. Originally published 2018 May 3. [Version 2] doi: 10.12688/f1000research.14819.2 (PMC6137411; doi:10.12688/f1000research.14819.2)
Supplement: Supplementary file 3 [file f1000research-7-17614-s0002.tgz › b2a79496-4ada-47c4-9682-a99ce867836d.zip!/Eight Iranian Health Journals/Journal of Knowledge and Health_9.docx]

© Journal of Knowledge and Health. This work is licensed under CC BY 4.0 (https://creativecommons.org/licenses/by/4.0/).

Volume & Issue: دوره 11، شماره 1: 1395

Title: بررسی ارتباط مقاومت آنتی‌بیوتیکی با agr تایپ در استافیلوکوک اورئوس مقاوم به متی‌سیلین جدا شده از زخم سوختگی بیماران بستری در سطح شهر تهران

http://knh.shmu.ac.ir/index.php/site/article/view/1199

مقدمه: استافیلوکوک عامل عمده عفونت‌هاي اكتسابي از بيمارستان و جامعه محسوب مي‌شود. اين باكتري واجد يك سیستم تنظیم‌کننده گلوبال (accessory gene regulator) است که در تنظیم تعداد بیشماری از عوامل کلونیزاسیون و بیماری‌زایی و مقاومت آنتی‌بیوتیکی نقش دارد. استافيلوكوك همچنين چهار پلی پپتید اصلي دارد كه متغیر بودن توالي‌هاي آن باعث می‌شود تا حداقل چهار گروه agr در استافیلوکوک اورئوس ايجاد شود. هدف اين مطالعه تعيين ارتباط الگوي آنتي‌بيوگرام با الگوي agr تايپ ايزوله‌ها مي‌باشد. مواد و روش‌ها: در اين مطالعه، شناسايي استافیلوکوک اورئوس مقاوم متی‌سیلین از زخم‌های سوختگی با روش فنوتيپي و ژنوتيپي انجام گردید. همچنين الگوی مقاومت به آنتی‌بیوتیک‌های تایجی سیلین (TGC)، سیپروفلوکساسین (CIP)، اریترومایسین (E)، کلوگزاسیلین (CX)، کلیندامایسین (CD)، ایمی پنم (IMI)، کوتریموکسازول (SXT)، کانامایسین (K)، تیکوپلانین(TEC) ، جنتامایسین (GM)، موپیروسین (MUP) و سفتریاکسون (CTR) نيز تعيين شد. agr تایپ‌ها با روشagr -locus PCR and ScaI RFLP  انجام شد و با استفاده از نرم‌‌افزارSPSS  ارتباط مقاومت آنتی‌بیوتیکی با agr تایپ مشخص شد. نتایج: در اين مطالعه تعداد 76 جدایه MRSA بررسي گردید. الگوی agr تایپ‌ها به‌ترتیب 6/75% تایپ I، 2/8% تایپ II، 4/5% تایپIII  و 8/10% تایپ IV بود. مقاوم‌ترین agr تایپ نسبت‌به آنتی‌بیوتیک‌ها مربوط‌به تایپ I می‌باشد. از نظر آماری ارتباط معنی‌داری بین هر agr تایپ با آنتی‌بیوتیک‌ها مشاهده نشد و فقط بین آنتی‌بیوتیک‌های CX،E ،CTR ،SXT ،GM ،CIP  و کلیه تایپ‌های agr رابطه معنادار وجود داشت (05/0P≤). نتیجه‌گیری: ارتباط معناداري بین هر agr تایپ و مقاومت آنتی‌بیوتیکی مشاهده نشد ولی مشاهده ارتباط معنی‌دار بین مقاومت تعدادی از آنتی‌بیوتیک‌ها با کلیه تایپ‌های agr احتمالاً مربوط‌به تعداد و منبع جداسازی باکتری یا مصرف بیش از اندازه این آنتی‌بیوتیک‌ها می‌باشد با‌توجه به اینکه لوکوس agr جزو ژن‌های بالا دستی می‌باشد لذا ممكن است با به‌کارگیری سیستم كروم سنسينگ بیشترین مقاومت دارویی را ایجاد کند.

Title: مقایسه هوش هیجانی، عزت‌نفس و ناگویی خلقی در زنان با افسردگی اساسی و زنان عادی در شهرستان شاهرود

http://knh.shmu.ac.ir/index.php/site/article/view/1055

مقدمه: اختلالات خلقی از جمله افسردگی اساسی از شایع‌ترین مشکلات روان پزشکی در جوامع مختلف به‌شمار می‌روند. هوش هیجانی به‌عنوان مؤلفه‌ای مهم، در سلامت و بهداشت روانی فرد تأثير بسیار مهم و ارزشمندی دارد و عزت‌نفس منبع حمایتی عمده‌ای است که افراد می‌توانند در مقابل رویدادهای منفی زندگی روزمره استفاده نمایند. از طرفی ناگویی خلقی مانع تنظیم هیجان شده و سازگاری فرد را دچار مشکل می‌نماید. پژوهش حاضر با هدف مقایسه هوش هیجانی، عزت‌نفس و ناگویی خلقی در زنان مبتلا‌به افسردگی اساسی و زنان عادی انجام گرفت. مواد و روش‌ها: طرح پژوهش از نوع علی مقایسه‌ای (پس رویدادی) بود. حجم نمونه آماری شامل 60 زن (30 نفر افسرده اساسی و 30 نفر عادی) که در بهار و تابستان 1393 به ساختمان پزشکان رسول اکرم (ص) و نور در شهرستان شاهرود مراجعه کرده بودند و با روش نمونه‌گیری در دسترس انتخاب گردیدند. ابزارهای سنجش شامل مقیاس هوش هیجانی شرینگ، عزت‌نفس کوپر اسمیت، ناگویی هیجانی تورنتو (TAS-20) و مقیاس افسردگی بک «BDI» بود. جهت تجزیه تحلیل داده‌ها از روش تحلیل واریانس چند متعیری (MANOVA) استفاده شد. نتایج: تحلیل یافته‌ها نشان داد که بین زنان افسرده اساسی و عادی در سطوح هوش هیجانی، عزت‌نفس و ناگویی خلقی تفاوت معناداری وجود دارد (001/0P<). نتیجه‌گیری: نتایج این پژوهش حاکی از ارتباط و اهمیت هوش هیجانی، عزت‌نفس و ناگویی خلقی در افسردگی است، که مي‌تواند در برنامه‌های پیشگیرانه و درمانی قابل استفاده باشد.

Title: نقش مشکلات تنظیم هیجان و ابعاد آن در پیش‌بینی رفتارهای خوردن آشفته در میان دانشجویان: تلویحاتی برای ارتقاء سلامت

http://knh.shmu.ac.ir/index.php/site/article/view/1077

مقدمه: مشکلات تنظیم هیجان، خوردن آشفته را پیش‌بینی می‌کند اما چگونه مشکلات تنظیم هیجان منجر به خوردن آشفته می‌شود، سؤالی است که بی‌جواب باقی‌مانده است. هدف از پژوهش حاضر، بررسي نقش مشکلات تنظیم هیجان و ابعاد آن در پیش‌بینی رفتارهای خوردن آشفته در میان دانشجویان بود. مواد و روش‌ها: این پژوهش به‌روش توصیفی از نوع همبستگی بر روی 264 نفر از دانشجویان دانشگاه علامه طباطبايی که با استفاده از برآورد جدول Morgan و به‌روش نمونه‌گیری خوشه‌ای چند مرحله‌ای انتخاب شدند، انجام گردید. برای جمع‌آوری داده‌ها از آزمون بازخورد خوردن و پرسشنامه تنظیم هیجان‌شناختی استفاده شد. داده‌ها با استفاده از محاسبه ضریب همبستگی پیرسون و رگرسیون گام به گام تحلیل شدند. سطح معنی‌داری، (01/0P<) در نظر گرفته شد. نتایج: نتایج نشان داد که بین مشکلات تنظیم هیجان و ابعاد آن (به استثنای بعد توجه مثبت مجدد) با رفتارهای خوردن آشفته همبستگی درونی معنی‌داری وجود دارد (01/0P<). همچنین، نتایج تحلیل رگرسیون گام به گام نشان داد که از میان ابعاد متغیر مشکلات تنظیم هیجان تنها ابعاد فاجعه‌پنداری، سرزنش دیگران و پذیرش توانستند در حدود 26% از واریانس خوردن آشفته را به‌طور معنی‌داری پیش‌بینی کنند (01/0P<). نتیجه‌گیری: نتایج پژوهش حاضر حاکی از اهمیت مشکلات تنظیم هیجان در پیش‌بینی رفتارهای خوردن آشفته می‌باشد و اين عامل می‌تواند ميزان بالايي از واریانس خوردن آشفته را تبيين نمايد.

Title: بررسی تأثير ماساژ افلوراژ بر شدت تنیدگی‌آورهای فیزیولوژیکی مرتبط با درمان در بیماران همودیالیزی

http://knh.shmu.ac.ir/index.php/site/article/view/842

مقدمه: علي‌رغم پیشرفت‌های زیاد در فناوری‌های درمانی، هنوز بسیاری از بیماران با تنیدگی‌آورهای فیزیولوژیک مرتبط با درمان همودیالیز مواجه هستند که سبب بروز عوارض جانبی در آنها می‌شود. مطالعه حاضر با هدف تعيين تأثير ماساژ افلوراژ به‌عنوان یک راهکار غیردارویی بر شدت تنیدگی‌آورهای فیزیولوژیکی مرتبط با درمان در بیماران همودیالیزی طراحي و اجرا گردید. مواد و روش‌ها: اين مطالعه از نوع کارآزمایی بالینی است که بر روی 41 بیمار تحت درمان با همودیالیز (20 نفر گروه آزمون) و (21 نفر گروه کنترل) در مرکز دیالیز بیمارستان امام خمینی تهران انجام گردید. ابزار گردآوری اطلاعات فرم اطلاعات دموگرافیک و پرسشنامه مقیاس تنیدگی‌آورها در بیماران همودیالیزی (HSS) بود. شدت تنیدگی قبل و طی مداخله و بعد از آن در هر دو گروه ثبت شد. براي بيماران گروه آزمون 12 جلسه (20 دقيقه‌ای) ماساژ درمانی انجام شد. تحليل داده‌ها با استفاده از نرم‌افزار  SPSSو آزمون‌هاي آماري من ويتني، آزمون علامت و آزمون فریدمن و همچنين كاي مجذور در سطح معني‌داري 05/0 انجام شد. نتايج: دو گروه از نظر ويژگي‌هاي پایه و دموگرافیک همگن بودند. در طی 4 هفته مداخله در گروه آزمون تنیدگی‌آور‌ها به‌تدریج کاهش یافت اما در گروه کنترل این تغییر مشاهده نشد، در پایان مداخله تمامی ‌ور‌ها در گروه آزمون کاهش یافت (05/0>P) در حالی که در گروه کنترل تغییر از لحاظ آماری معنادار نبود (05/0<P). همچنين در پايان هفته چهارم شدت تنیدگی‌آورها بین دو گروه تفاوت معني‌داري داشت (05/0>P). نتیجه‌گیری: ماساژ افلوراژ بر کاهش شدت تنیدگی‌آورهای فیزیولوژیکی ناشي از همودیالیز مؤثر است لذا پيشنهاد مي‌شود این روش بر روی طيف وسيع‌تری از بيماران دياليزي انجام گيرد تا اثر قطعی آن مشخص گردد.

Title: اثر ترکیبی تمرین استقامتی و دوزهای مختلف آتورواستاتین بر تغییر شکل ساختاری قلب پس از انفارکتوس میوکارد در موش‌های صحرایی نر

http://knh.shmu.ac.ir/index.php/site/article/view/1176

مقدمه: استاتین‌ها و فعالیت ورزشی هر دو دارای اثرات سودمندی در پیشگیری از بیماری‌های قلبی- عروقی هستند. با این حال، مصرف طولانی مدت داروهای استاتینی به‌ویژه در دوزهای بالا دارای اثرات جانبی نامطلوبی است. پژوهش حاضر با هدف بررسی اثر ترکیبی تمرین استقامتی و سه دوز داروی آتورواستاتین بر تغییر شکل ساختاری قلب پس از انفارکتوس میوکارد در موش‌های صحرایی نر صورت گرفته است. مواد و روش‌ها: 75 سر موش صحرایی نر نژاد ویستار ( با وزن 250-210 گرم) به‌طور تصادفی به 9 گروه کنترل کاذب (شم)، کنترل، تمرین استقامتی، آتورواستاتین (5، 10 و 15 ميلي‌گرم/کیلوگرم) و تمرین + آتورواستاتین (5، 10 و 15 ميلي‌گرم/کیلوگرم) تقسیم شدند. انفارکتوس میوکارد به‌وسیله تزریق زیرجلدی ایزوپرنالین (mg/kg 150) در دو روز متوالی القاء شد. مداخله دارویی و تمرین استقامتی 2 روز پس از انفارکتوس میوکارد شروع و به مدت 4 هفته ادامه یافت. جهت بررسی آسیب نکروزی و میزان بافت فیبروزی به‌ترتیب از رنگ‌آميزي هماتوکسیلین- ائوزین و تری کرم ماسون استفاده شد. نتایج: ترکیب تمرین استقامتی و دوزهای مختلف داروی آتورواستاتین باعث کاهش معنی‌دار میزان آسیب نکروزی و بافت فیبروزی در مقایسه با گروه کنترل گردید (01/0>P). تمرین ورزشی استقامتی به تنهایی تغییر معنی‌داری در میزان آسیب نکروزی ایجاد نکرد اما باعث افزایش معنی‌دار بافت فیبروزی (001/0>P) در مقایسه با گروه کنترل گردید. دوزهای مختلف داروی آتورواستاتین به تنهایی باعث کاهش معني‌دار میزان آسیب نکروزی در مقایسه با گروه کنترل گردید (001/0>P)، اما اختلاف بین این گروه‌ها و گروه کنترل از نظر میزان بافت فیبروزی تنها در دوز 15 ميلي‌گرم/کیلوگرم از لحاظ آماری معني‌دار بود (001/0>P). نتیجه‌گیری: نتایج این مطالعه نشان داد که ترکیب تمرین استقامتی و دوزهای مختلف داروی آتورواستاتین در بهبود شاخص‌های آسیب قلبی ناشی از انفارکتوس میوکارد مؤثرتر از تمرین و دارو به تنهایی مي‌باشد. با این حال، استفاده از تمرین استقامتی در کنار درمان دارویی نمی‌تواند باعث کاهش دوز مصرفی آتورواستاتین گردد.

Title: بررسی عملکرد جاذب لیکای اصلاح شده (TiO2/ LECA) در حذف سورفاکتانت آنیونی از فاضلاب

http://knh.shmu.ac.ir/index.php/site/article/view/1093

مقدمه: سورفاکتانت‌ها يا عوامل فعال سطحي مواد شيميايي آلي هستند که با تجمع در سطح مشترک گاز- مايع يا جامد- مايع، خصوصيات آب را تغيير داده و با کاهش کشش سطحي آب، عمل پاک‌کنندگي را تسريع مي‌كنند. با تخليه مستقيم فاضلاب خام يا پساب‌هاي تصفيه ‌شده، دترجنت‌ها به محيط وارد شده و با ايجاد آلودگي، سبب کاهش کیفیت در منابع آب و افزایش هزينه‌ها مي‌شوند. هدف پژوهش ارزیابی عملکرد لیکای حاوی نانو ذره دی‌اکسید تیتانیم (TiO2) در حذف سورفاکتانت از فاضلاب بود. مواد و روش‌ها: اين مطالعه يك تحقیق تجربی است كه در آن به‌منظور آماده‌سازي جاذب لیکا جهت افزایش میزان کارآیی و خاصیت کاتالیستی، از دی اکسید تیتانیم (Tio2) استفاده شد. تأثير شاخص‌هاي pH (3، 5، 7، 9 و 11)، مقدار جاذب (g/L 25/0-2)، غلظت اوليه سورفاکتانت ( mg/L25-200) و زمان تماس (min 15-120) توسط جاذب لیکای اصلاحي مورد بررسي قرار گرفت. آزمایش‌ها بر روی نمونه سنتتیک، سپس در شرایط بهینه بر روی نمونه واقعی فاضلاب نیز انجام شد. اندازه‌گیری غلظت سورفاکتانت، با دستگاه اسپکتروفتومتر انجام شد. نتايج: حداکثر حذف سورفاکتانت توسط لیکای اصلاح شده از محلول‌های سنتتیک سورفاکتانت، در شرایط بهینه (زمان تماس 30 دقیقه و pH برابر 5، مقدار جاذب 1 گرم بر لیتر، غلظت اولیه سورفاکتانت 100 ميلي‌گرم بر لیتر) به میزان 98 درصد و در همین شرایط در نمونه‌های واقعی 87 درصد حاصل شد. نتایج به‌دست آمده از مطالعات ايزوترمي نشان داد كه حذف سورفاکتانت از معادله ایزوترم فروندلیخ (973/0R2 =) تبعیت دارد. همچنین معادله شبه درجه دوم بهترين سازگاري را با داده‌های سینتیکی دارد. نتیجه‌گیري: نتایج مطالعه نشان داد که لیکای حاوی نانوذرات دی اکسید تیتانیم مي‌تواند به‌عنوان جاذبی مؤثر جهت حذف سورفاکتانت آنیونی از محلول‌های آبی به‌کار رود.

Title: بررسي ميزان آلودگي باكتريايي آب يونيت‌هاي كلينيك‌هاي دندانپزشكي شهر شاهرود در سال 1393

http://knh.shmu.ac.ir/index.php/site/article/view/1132

مقدمه: از آنجايي كه اعضاء تيم دندانپزشكي و بيماران در معرض آب و آئروسل‌هاي ايجاد شده از يونيت دندانپزشكي قرار مي‌گيرند، بررسي كيفيت آب يونيت دندانپزشكي از اهميت قابل توجهي برخوردار مي‌باشد. هدف از اين مطالعه بررسي ميزان آلودگي باكتريايي سيستم‌هاي آبي يونيت‌هاي كلينيك‌هاي دندانپزشكي شاهرود بود. مواد و روش‌ها: اين مطالعه توصيفي- تحليلي به روش سرشماري در سال 1393 بر روي آب يونيت كليه مطب‌ها و كلينيك‌هاي دندانپزشكي شهر شاهرود انجام شد، 560 نمونه آب از 4 قسمت يونيت شامل پوار آب و هوا، مجراي سرتوربين قبل و بعد از فلاشينگ، ليوان پر كن و 2 نمونه آب از منبع آب شهري ورودي به يونيت‌ها گرفته شد. نمونه‌گيري در روز شنبه و پنج‌شنبه قبل از شروع كار و بعد از اتمام كار صورت گرفت. نمونه‌گيري با استفاده از ظروف استريل صورت گرفته و به آزمايشگاه ميكروبيولوژي منتقل شد. تمام نمونه‌ها در محيط بلادآگار و مك كانكي آگار به مدت 72 ساعت در دماي C°37 انكوبه شدند، سپس كلني‌هاي رشديافته شمارش شدند. ضمناً تمامی نمونه‌ها در محیط کشت اختصاص يل ژيونلا) BCYE حاوي آنتي‌بيوتيك‌هاي پليميكسين B و نكومايسينوسيكلوهگزاميد) درجار شمعدار در 37 درجه سانتي‌گراد و رطوبت 70-90 درصد انكوبه شدند. داده‌ها با استفاده از نرم‌افزار SPSS و آزمون آماري ANOVA و آزمون t مستقل مورد تجزيه و تحليل آماري قرار گرفت و ميزان 05/0P< معني‌دار تلقي گرديد. نتايج: کلیه نمونه‌ها از نظر وجود باکتری لژیونلا منفی بودند. میانگین شمارش باکتری‌ها cfu/ml4671 بود میانگین شمارش باکتری‌ها روز چهارشنبه (cfu/ml3860) کمتر از آلودگی در روز شنبه (میانگین cfu/ml6320) بود اما براساس آزمون آماری ) t142/0P=) اختلاف بین نتایج معنی‌دار نبود، همچنين میانگین شمارش باکتریایی به تفکیک زمان نمونه‌گیری، میزان آلودگی بعد از کار (cfu/ml6250) بيشتر از قبل از شروع کار (cfu/ml4050) بود (186/0P=). بیشترین ميانگين شمارش باکتری‌ها مربوط‌به مطب‌های بخش خصوصی و کمترین میانگین مربوط‌به درمانگاه خیریه بود (01/0P<). نتيجه‌گيري: نتایج این مطالعه نشان داد که میزان آلودگی آب یونیت‌های دندان پزشکی بالا می‌باشد و دندانپزشکان باید همواره به کنترل میکروبی آب یونیت‌های دندانپزشکی توجه داشته باشند تا به ارتقاء سلامت خود، بیماران و پرسنل مطب کمک نمایند.

Title: اثر دو شیوه تمرینی شنای استقامتی همراه با مصرف ویتامین C بر شاخص‌های آنمی موش‌های صحرایی نر ویستار

http://knh.shmu.ac.ir/index.php/site/article/view/1138

مقدمه: نشان داده شده است که تمرینات طولانی مدت شنا موجب آنمی مي‌شود. بنابراین هدف از این مطالعه، ارزیابی تأثير مصرف مکمل ویتامین C بر شاخص‌های آنمی پس از تمرینات شنا زیربیشینه و بیشینه در موش‌های صحرایی نر ویستار بدون کمبود آهن بود. مواد و روش‌ها: در این مطالعه 60 سر موش صحرایی نر ویستار با دامنه وزنی 170 تا 190 گرم و با میانگین سنی 6 تا 8 هفته‌ای به‌طور تصادفی در گروه‌های 1: کنترل (10Con, n=) 2: کنترل+ ویتامین C (10Con+C, n=) 3: تمرین زیربیشینه (10S, n=) 4: تمرین زیربیشینه + ویتامین C (10S+C, n=) 5: تمرین بیشینه (10M, n=) و 6: تمرین بیشینه + ویتامین C (10M+C, n=) تقسیم شدند. تمرینات شامل 10 هفته شنا با دو شدت متوسط و بیشینه (5 روز در هفته) بود. تمرینات زیر بیشینه به مدت 1 ساعت و تمرینات بیشینه به مدت 3 ساعت در هر جلسه انجام شد. گروه‌هایی که ویتامین C مصرف مي‌کردند، 100 ميلي‌گرم ویتامین C در 100 میلی‌لیتر آب آزمودنی‌ها حل شد. برای اندازه‌گیری سطوح سرمی شاخص‌هاي آنمي، آهن سرم، فریتین، رتیکولوسیت، هموگلوبین (Hb)، هماتوکریت (Hct)، سلول‌های قرمز خون (RBC)، ظرفیت تام اتصال آهن (TIBC)، میانگین هموگلوبین سلول (MCH)، میانگین غلظت هموگلوبین سلول (MCHC) و میانگین حجم گلبول‌های قرمز (MCV)، 5 سی سی خون از ورید اجوف تحتانی گرفته شد. نتايج: تجزيه و تحليل آماری داده‌ها نشان داد که میزان آهن سرم، فریتین، TIBC و MCV در بین گروه‌های مورد مطالعه تفاوت معناداری نداشت. میزان رتیکولوسیت‌ها در گروه Con+C و گروه S+C نسبت‌به سایر گروه‌های دیگر به‌طور معناداری کاهش یافت. در همین راستا میزان RBC و هماتوکریت نیز در گروه S و S+C نسبت‌به سایر گروه‌های دیگر کاهش یافت، اما میزان هموگلوبین در گروه S افزایش پیدا کرد. همچنین میزان MCH و MCHC در گروه‌های S و S+C نسبت‌به سایر گروه‌های مورد مطالعه به‌طور معناداری افزایش پیدا کرد. نتیجه‌گیری: ده هفته تمرینات شنای زیربیشنه موجب افزایش هموگلوبین، MCH و MCHC مي‌شود، اما تأثيری بر میزان آهن، فریتین و TIBC نداشت. از طرفی تمرینات شنای با شدت بالا تأثيری بر شاخص‌های آنمی نداشت. همچنین مصرف محلول 1/0 ویتامین C نیز همراه با تمرینات شنای بیشینه و زیربیشینه علیرغم افزایش غیرمعنادار فریتین تأثيری بر وضعیت آهن در موش‌های صحرایی نر ویستار نداشت.

Title: مقايسه تأثير نیکوتینیک اسید و سولامر بر فسفر بالای سرم در بیماران همودیالیزی: یک مطالعه کارآزمایی بالینی دوسویه کور

http://knh.shmu.ac.ir/index.php/site/article/view/756

مقدمه: کنترل میزان فسفر در بیماران دیالیزی بسیار مهم و ضروری بوده که به طرق مختلف انجام می‌شود. هدف اين مطالعه مقايسه اثر نیکوتینیک اسید ( نیاسین) و سولامر بر سطح فسفر بيماران همودياليزي مي‌باشد. مواد و روش‌ها: اين تحقيق يك مطالعه كارآزمايي باليني بوده كه بر روي 120 بيمار دیالیزی که به دو گروه 60 نفره مورد (مصرف نیاسین) و شاهد (مصرف سولامر) تقسیم شدند، پس از اخذ رضایت‌نامه آگاهانه انجام شده است. دوز نیاسین از 200 به 600 ميلي‌گرم در روز و دوز سولامر از 400 به 1200 ميلي‌گرم در روز به‌صورت ماهانه و طي سه مرحله افزايش يافت. آزمايشات مربوط‌به سطح فسفر در هر دو گروه انجام و نتایج دو گروه به كمك آزمون‌هاي آماري مرتبط با هم مقايسه خواهد شد. نتایج: از بررسي كل بيماران مشخص شد كه ميانگين سني بيماران در گروه مورد 3/21± 5/51 سال و گروه شاهد 5/22±7/50 بود كه تفاوت معني‌داري با هم نداشتند. در این تحقیق ميزان فسفر در شروع مطالعه در گروه مورد 37/1 ±67/6 ميلي‌گرم بر دسي‌ليتر و در گروه شاهد 95/0 ±77/6 ميلي‌گرم بر دسي‌ليتر بود كه تفاوت معني‌داري با هم نداشتند. همچنين مشخص شد كه به‌طور معني‌داري استفاده از نياسين باعث كاهش فسفر (001/0P<) سرم گشته و با افزایش دوز مصرفی آن، حتی تأثير کاهندگی فسفر خون آن، از سولامر هم بیشتر مي‌شود. نتيجه‌گيري: نتایج این تحقیق نشان داد که نيكوتينيك اسيد همانند سولامر باعث كاهش فسفر در بيماران همودياليزي می‌شود ولی لازم است براي تعيين دوز مناسب دارو مطالعات کامل‌تری انجام شود.

Title: بررسی تأثیر مداخله آموزشی براساس الگوي اعتقاد بهداشتي بر رفتارهای ‌پيشگيري‌كننده از خشونت خانگی در دانش‌آموزان دختر دوره دوم متوسطه شهر تهران

http://knh.shmu.ac.ir/index.php/site/article/view/1302

مقدمه: اين مطالعه با هدف تعیین تأثیر مداخله آموزشی براساس الگوي اعتقاد بهداشتي بر رفتارهای پیشگیری‌کننده از خشونت خانگی بين دختران دانش‌آموز دوره دوم متوسطه شهر تهران انجام شده است. مواد و روش‌ها: 242 دانش‌آموز دختر شهر تهران به‌صورت تصادفی به دو گروه مداخله و كنترل تقسیم شدند. ابزار گردآوري داده‌ها پرسشنامه‌ شامل اطلاعات دموگرافیک و سازه‌های مدل اعتقاد بهداشتی است. قبل از مداخله آموزشي، پيش آزمون انجام شده و سپس براساس نتایج حاصله يك برنامه آموزشي در زمينه خشونت خانگي براي گروه مداخله اجرا گرديد. 3 ماه بعد از مداخله همان پرسشنامه مجدداً به افراد مورد مطالعه داده شد تا تكميل نمايند. داده‌ها با استفاده از نرم‌افزار SPSS و آزمون‌هاي آماري کای- اسکوئر، ویلکاکسون و من ویتنی تجزيه و تحليل گرديد. نتایج: نمرات آگاهي (324/0=P)، نگرش (05/0P>) و عملكرد (758/0=P) در دو گروه مداخله و كنترل قبل از آموزش تفاوت معني‌داري نداشت اما بعد از آموزش نمرات آگاهی و در ابعاد الگوي اعتقاد بهداشتي نمرات حساسیت درك شده، شدت درك شده، منافع درك شده، موانع درک شده و عملکرد در دانش‌آموزان بين دو گروه مداخله و كنترل اختلاف معني‌داري مشاهده شد (001/0P<) ولي در بعد راهنما براي عمل (837/0=P) و خودكارآمدي (262/0=P) اين اختلاف از نظر آماري معنادار نبود. نتيجه‌گيري: نتایج این مطالعه بیانگر تأثیر آموزش از طریق مدل اعتقاد بهداشتی بر افزایش آگاهی، ادراکات (‌نگرش) و عملکرد دانش‌آموزان در مورد رفتارهای پیشگیری‌کننده از خشونت خانگی مي‌باشد.
